# Marjolin's Ulcer After Saphenectomy: A Case Report

**DOI:** 10.7759/cureus.30912

**Published:** 2022-10-31

**Authors:** Carlos E Rodriguez, Diego F Cardona, Theresa W Rodriguez, Sophia Garcia, William F Willmer

**Affiliations:** 1 General Surgery, Instituto de Pós Graduação Médica Carlos Chagas, Rio de Janeiro, BRA; 2 General Surgery, Insituto de Pós Graduação Médica Carlos Chagas, Rio de Janeiro, BRA; 3 Surgical Oncology, Hospital Universitário Pedro Ernesto (HUPE) Universidade do Estado do Rio de Janeiro (UERJ), Rio de Janeiro, BRA

**Keywords:** treatment, chronic wounds, squamous cell cancer, ­skin cancer, marjolin’s ulcer

## Abstract

Chronically injured areas have the possibility of transforming into malignant tissue, with squamous cell carcinoma being the most common type. This rare entity is known as Marjolin's ulcer. Most of these ulcers derive from chronic burn wounds. This case report exhibits a rare Marjolin’s ulcer that developed on a 50-year-old male with a previous saphenectomy on his left leg. The patient was brought to the operating room (OR), for excision of the ulcer with a rotation flap to correct the defect. There is still no definite treatment protocol for Marjolin’s ulcer. In the present article, the most common treatments are discussed. The main takeaway of this case is the prevention of Marjolin’s ulcer by timely treating ulcerative lesions.

## Introduction

Marjolin's ulcer was named after the French surgeon Jean Nicholas Marjolin, who first described the condition of the lesion in 1828. The term is given to aggressive squamous cell carcinoma that arises in chronically injured areas, burn scars being the most common [1]. It is a rare entity, occurring in around 1-2% of scar tissue [2]. It is unknown why a chronically injured area undergoes malignant transformation and becomes a Marjolin’s ulcer. Some theories include immunosuppression, repeated trauma that causes cell atypia, and obliteration of local lymphatic drainage due to avascularity in the area [3]. The current standard diagnosis for a Marjolin’s ulcer focuses on patient history and histologic analysis of the lesion via biopsy. Wound surveillance on any wound that doesn’t heal is recommended to avoid malignant progression [4]. Nowadays there’s still no definite treatment protocol for this entity, nevertheless, the most common treatment is based on surgical excision, amputation, or Mohs surgery and skin grafting or flapping [3]. The objective of this article is to present a rare case of a Marjolin’s ulcer on a male patient with a saphenectomy wound and its treatment.

## Case presentation

A 71-year-old male, skin phototype II of the Fitzpatrick scale, arrived at our surgical department with an ulcerative lesion on the left lower limb in the tibial region. The patient had a history of coronary bypass surgery a year prior. The donor site for the surgery developed a non-healing lesion. He came to our department in February 2022, after an external consultation, with a biopsy positive for squamous cell carcinoma. Following examination, the patient was found to have an oval-shaped ulcer on the left tibial region, which was approximately 8x6 cm. The wound granulation exhibited a cauliflower-like pattern, with a thick layer of necrotic material. A large amount of exudate was observed on the wound. He didn’t have any enlarged lymph nodes. The patient refused to get computed tomography or magnetic resonance imaging to rule out any metastases or bone invasion. With the biopsy, characteristics of the lesion, and history of saphenectomy he was diagnosed with Marjolin’s ulcer.

**Figure 1:**
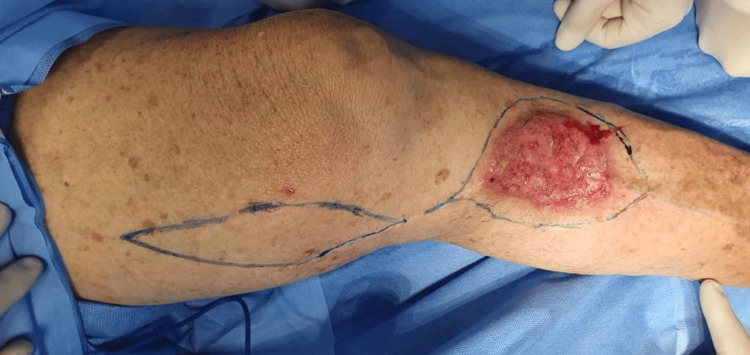
Pre-operative wound Marking of excision borders and flap donor area before surgical repairment

The chosen treatment was surgical excision. During the pre-operative period, the patient had no relevant laboratory or neurovascular alterations, and, his treatment with clopidogrel was interrupted. To initiate the procedure, the excision borders and the flap area for donor tissue were marked, as shown in the first image (Figure 1). 

**Figure 2:**
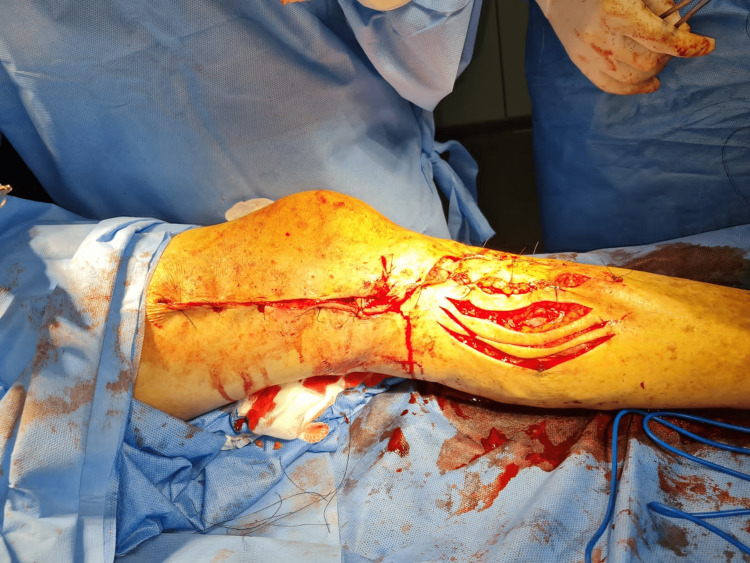
Intraoperative view of the repaired wound Intraoperative view of the lesion covered with pedicled muscle.

Afterward, a dissection of the serrated lesion was performed, leaving margins of 2 cm of healthy tissue, and exposing the left tibial bone. To cover the wound, a flap rotation was performed with pedicled muscle (Figure 2). 

No lymph node resection was carried out. The patient remained hospitalized for seven days after surgery with an uneventful recovery and had his dressing changed every day. He was scheduled for regular follow-ups for five years.

## Discussion

Squamous cell carcinoma is a common disease that accounts for 20% of non-melanoma skin cancers. Its cases have increased in recent years mainly due to increased sun exposure, tanning beds, population aging, and improved diagnostic methods. Squamous skin cancer can occur anywhere on the skin, including the head and neck (55%), back of hands (18%), legs (13%), arms (3%), back of back (4%), and abdomen or chest (4%). The tumor may initially appear as chronic ulceration and nodules that progress. Other manifestations include sores with excessive granulation tissue, that rapidly increase in size and bleed to the touch [5]. The development of squamous skin cancer in areas that are not exposed to the sun is less common, but it represents the most common subtype in people with dark skin. In black individuals, common sites of squamous skin cancer are legs, anus, areas of chronic inflammation, or scars. Lesions in scar areas account for 20 to 40% of all cases in these patients [6].

From a clinical point of view, two types of carcinomas are distinguished: ulcerative; with infiltrated lesions, raised edges, and surrounding induration, and less frequently, exophytic; papillary lesions resembling granulation tissue. Secondary burn scars may undergo chronic and recurrent ulceration before the malignant transformation. Pain could be an indication of malignancy. Other accompanying symptoms may be the presence of bleeding, exudate, or foul odor [7]. The tumor usually starts at one edge of the ulcer and grows slowly. This focal nature of the malignancy increases the risk of false negatives with biopsy, recommending multiple incisional or excisional biopsies [8]. Carcinomas that arise in areas of scarring are typically aggressive and associated with a worse prognosis [9].

The risk of disease recurrence or metastases is approximately 20 to 30% and is related to histological grade and tumor size [3,10]. The most frequent sites of metastases are regional lymph nodes, but they can also occur in the lung, liver, brain, skin, or bones. Clinical findings strongly suggest the diagnosis, but histopathological examination is necessary for its confirmation. During the biopsy procedure, the need to extend the biopsy up to the reticular dermis layer must be taken into account in order to pinpoint tumor invasion. Superficial biopsies are reserved for initial lesions without evidence of invasion. When the diagnosis is made, patients with squamous skin carcinoma should be examined very carefully, including palpation of possible lymphadenopathy and other signs of metastases [8].

The most accepted treatment for Marjolin's ulcer consists of complete removal of the tumor with wide safety margins of at least 2 cm, covering the defect with grafts or flaps. Grafting is recommended as the first technique for closing the defect to avoid hidden recurrences, leaving a one-year latency to cover the area with a local or distant flap. Amputation is reserved for when there is the involvement of joint spaces, bone invasion, or extensive deep local invasion. Lymph node dissection is controversial. If there are clinically palpable lymph nodes or the histological examination of the primary skin ulcer indicates a high degree of malignancy, lymph node dissection would be indicated; however, based on the high percentage (53.8%) of lymph node metastases in lower limb ulcers, with a poor prognosis, some authors advocate elective lymph node dissection [3].

As for the evolution of Marjolin's ulcer, the acute form has a good prognosis, while patients with long latency periods have metastasis and a mortality rate of 30% [8]. Tumors in the head, neck, and upper extremities progress better than those in the trunk and lower extremities. As previously mentioned, the lower extremities are the site with the highest risk of metastasis (50-54%) and with the lowest 5-year survival percentages [7]. Well-differentiated exophytic tumors are associated with a better prognosis than infiltrated, ulcerated, and poorly differentiated forms. The most important prognostic indicator is the presence of regional lymph node metastases. The percentages of metastases to regional lymph nodes in Marjolin's ulcer range from 34.8 to 36%, being significantly higher than in squamous skin carcinoma secondary to actinic injury (0.5-16%) [6]. The lack of peritumoral T lymphocyte infiltration is significantly associated with an increased risk of metastasis. Thus, in cases of Marjolin's ulcer with significant peritumoral infiltrate, survival, without recurrence or metastasis, is higher, regardless of the degree of tumor differentiation, while in tumors with little or no infiltrate, even well-differentiated, there is a higher risk of metastasis. As cases of T lymphopenia preceding or accompanying the development of metastases have already been described, the use of immunotherapy with thymic factor (thymostimulin) was proposed as post-surgical therapy [8].

## Conclusions

In conclusion, this case report highlights the importance of surveillance in any wound that becomes chronic to avoid Marjolin’s ulcer formation. Biopsy should be considered on any wound that does not heal in a year to rule out malignancy. Early stages of Marjolin’s ulcers are easier to treat and pose less metastasis danger and better prognosis than advanced cases. On the other hand, the prognosis in patients with larger ulcers is mostly reserved, as they can become malignant and are characterized by the recurrence of previous scars. Therefore, timely treatment once the entity is identified, and close follow-up for the years ahead are necessary to ensure the patient’s well-being. Nowadays it is well-known that the use of auxiliary reconstructive procedures obtains good results in these cases.
